# Establishment of a new prognostic risk model of GNG7 pathway-related molecules in clear cell renal cell carcinoma based on immunomodulators

**DOI:** 10.1186/s12885-023-11265-8

**Published:** 2023-09-13

**Authors:** Jun Zheng, Weili Zhang, Junyong Zhang

**Affiliations:** Department of Urology, the Second Affiliated Hospital of Chongqing Medical University, Chongqing, 400000 China

**Keywords:** Immune infiltration, GNG7, Clear cell renal cell carcinoma, Prognosis

## Abstract

Clear cell renal cell carcinoma (CCRCC) is a common tumor of the urological system for which surgery is the preferred treatment, but there is a lack of therapeutic options after surgery. This study aims to explore the biological role of GNG7 on CCRCC from a genetic perspective. Differences in mRNA expression and patient survival of GNG7 in patients with CCRCC and healthy patients were analyzed using the TCGA database. It was observed that GNG7 gene expression was downregulated in CCRCC tissue compared with healthy tissue, and high GNG7 predicted better prognosis for patients, and GNG7 also showed strong variability in clinical and TMN staging. The immune relevance of GNG7 and related genes was explored using renal cancer data from CCLE and TISIDB database. It was verified that the risk score constructed by 7 GNG7-related regulators might be used as an independent prognostic risk factor for CCRCC. A CCRCC prognostic model that involved 7 immune genes was further established to predict the survival probabilities of patients. At last, the GEO database and immunochemical tissue staining were used to validate GNG7 expression in CCRCC. Our study proposed a novel panel of genes to predict CCRCC OS based on GNG7-related immune genes, which may help to accurately predict the prognosis of CCRCC patients and make better clinical decisions for individual treatment.

**Supplementary Information:**

The online version contains supplementary material available at 10.1186/s12885-023-11265-8.

## Introduction

Renal cancer is a common urinary system tumor [1]. Due to the lack of obvious clinical features in the early stage of renal cancer, it is impossible to make a more accurate early diagnosis of the disease. Surgical treatment is the preferred choice for renal cancer, since it does not show satisfactory sensitivity to chemotherapy and radiotherapy [2, 3]. Typically, clear cell renal cell carcinoma (CCRCC) is the most important tissue subtype of renal cancer, accounting for 80% of all cases [4]. CCRCC patients are associated with higher mortality and metastasis rates [5]. Therefore, improving the survival rate of CCRCC patients after surgical treatment has always been a problem for urologists. Fortunately, with the advancement in knowledge of biology and comprehensive treatment of CCRCC, tumor immunotherapy has gradually become a promising alternative treatment approach for some urinary tract tumors due to its high accuracy and safety [6–8]. The immune system can generate anti-tumor immunity by affecting tumor growth and mutation behaviors. Meanwhile, tumor cells may damage immune cells in different ways [9, 10]. The efficacy of immunotherapy in urinary system tumors has been confirmed [11, 12]. However, similar to other therapies, only a fraction of patients with urinary tract tumors can benefit from immunotherapy [13–15]. Some evidence suggests that tumor-infiltrating white blood cells are related to clinical efficacy and cancer prognosis, including CCRCC [16, 17]. However, the molecular characteristics of tumor immune microenvironment (TIME) still need to be explored in depth. Therefore, it is necessary to comprehensively understand the immunology and molecular regulatory mechanisms of CCRCC, so as to ensure the success of immunotherapy.

GNG7 belongs to the large family of G proteins [18]. In the mating pheromone response pathway, G-protein-mediated growth arrest induced by cell contact has been confirmed, while GNG7 may be involved in this process to prevent growth arrest in multicellular organisms and control cell proliferation [19]. According to this hypothesis, cells exposed to GNG7 will stop further proliferation and start the differentiation process mediated by G protein signals. On the other hand, GNG7 is a promising therapeutic target for cancer. Decreased GNG7 expression has been detected in multiple tumors, including head and neck squamous epithelial tumors, breast cancer (BC), gastric cancer (GC), kidney cancer, colorectal cancer (CRC) and lung adenocarcinoma (LUAD) [19–23]. So far, the mechanism supporting the carcinogenic effect of down-regulated GNG7 expression in CCRCC remains largely unclear. In previous reports, genes and methylation are suggested to be related to GNG7’s effect on suppressing tumor progression [19, 24]. However, up to now, no existing study has reported whether GNG7 affects the occurrence and progression of tumor diseases by participating in the immune system.

As for the early detection of tumors, the previously reported techniques include the more costly conventional assays such as protein blotting, immunohistochemistry and reverse transcription-polymerase chain reaction (RT-PCR) [25–27]. In recent years, the development of low-cost biosensors for cancer markers has increased rapidly due to the rapid improvement in bioanalytical technology that has made it possible to explore large-scale data [28]. Some studies have indicated that the use of cell surface sialic acid-rich glycoproteins to detect kidney cancer cells in human urine can be better used for the diagnosis of CCRCC and is well compatible in metastatic kidney cancer [29]. In future research efforts, the development and selection of biosensors and biomarkers will definitely be a trend for early cancer diagnosis.

Based on previous studies, there is a reasonable assumption that GNG7 may exert a suppressive role in certain pathogenic aspects of tumors. Our hypothesis posits that GNG7, serving as a potential biomarker in clear cell renal cell carcinoma (CCRCC), could significantly influence the oncogenesis and progression of tumor disease by actively participating in the regulation of the immune system. To test this hypothesis, we utilized the TIMER database to investigate the relationship between GNG7 and immune cells in CCRCC. Subsequently, we carefully selected GNG7-related genes from CCLE kidney cancer cell lines and performed Gene Ontology (GO) and Kyoto Encyclopedia of Genes and Genomes (KEGG) analyses to explore the immune pathways associated with GNG7.

To further validate our findings, we developed a CCRCC prognostic model based on seven GNG7-related immunomodulators. This model's feasibility was subsequently verified through multi-factor regression analysis, using GNG7-related immunomodulators from the TISIDB database.

**Fig. 1:**
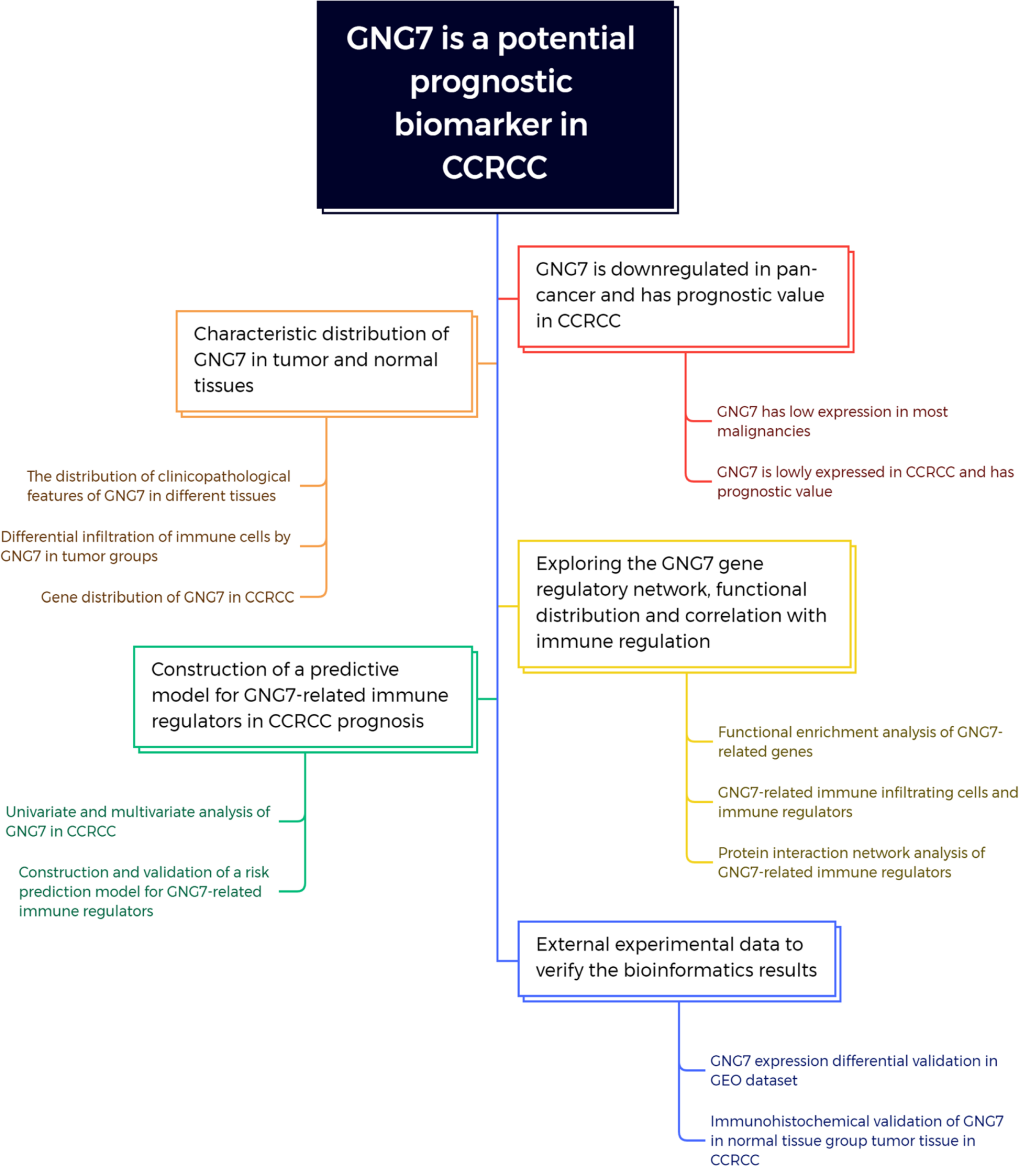
Workflow for downstream analysis

Through our ongoing study, we aim to discuss the immunological relevance of GNG7 in CCRCC, construct a clinical prognostic model, and identify novel immunotherapeutic targets for CCRCC. We believe that these investigations will contribute to a deeper understanding of the role of GNG7 in tumor-immune interactions, potentially leading to the development of more effective personalized treatment strategies for CCRCC patients. The detailed flow chart of this study is shown in Fig. 1.

## Methods and materials

### TCGA Datasets

TCGA is a cancer and tumor gene mapping project initiated by the U.S. government. It utilizes genome analysis technology, especially for the large-scale genome sequencing, to map the genome mutations of all human cancers and conduct systematic analysis, so as to improve people’s scientific understanding towards the molecular basis of cancer pathogenesis and improve our ability to diagnose, treat and prevent cancer. The CCRCC dataset was obtained from TCGA project. All the RNA-seq transcriptome data and clinical features of 539 cancer tissues and 72 healthy tissues were included. Meanwhile, the selected samples also contained GNG7 gene expression profiles and related clinical information, including age, gender, TNM stage, and grade [30]. The LIMMA software package in R software was used to process the above data [31]. GSE40435(normal:101,tumor:101) was downloaded from GEO database and used for differential analysis validation.

## Clinical Proteomic Tumor Analysis Consortium (CPTAC) and UALCAN

CPTAC is a mass spectrometry-based proteomics database that provides protein data for 7 different cancer types. Here, the protein expression of GNG7 was analyzed in detail from CPTAC through UALCAN, an online web resource used to analyze the publicly available cancer data [32].

## Detection of tumor infiltrating immune cells in renal cell carcinoma

CIBERSORT is an online data processing website, which uses RNA-Seq data to analyze the immune infiltration profiles of cells through linear support vector regression. After extracting the characteristics of immune cells, the proportions of various cell components in Bulk-seq were reversed on this basis [33]. In the CIBERSORT method, cell components are calculated using the principle of deconvolution, usually linear deconvolution. This method can be used to identify and quantify a total of 22 different types of immune cells in tissues.

## GNG7 and tumor immune infiltration

Tumor Immune Evaluation Resources is an online website that can perform detailed analysis and evaluation of immune cell infiltration status in pan-cancer. The website provides three modules for immunization, exploration, and evaluation to investigate the association between immune infiltration and genetic or clinical characteristics, and four modules to explore the association with cancer in TCGA cohort. Each module can generate a functional heat map. The infiltration profiles of six immune cells and four immunosuppressive cells of GNG7 in CCRCC were obtained through this online tool [34].

## Gene set enrichment analysis (GSEA)

Cancer Cell Line Encyclopedia (CCLE) is a database developed to investigate the expression of multiple omics in tumor cell lines. The database contains 1457 cell lines and relevant data on the expression profiles, copy numbers, and methylation of genes in the cell lines. CCLE allows to visually map genes expressed in tumors from the perspective of cell lines, including cell line distribution, mutation data, and CpG methylation. In this study, the RNA-seq data of 32 renal cancer cell lines were downloaded and processed from the CCLE online database. After processing and filtering data with P ≥ 0.05, 207 genes significantly related to GNG7 gene were finally obtained. With a limit of 50 genes, a correlation heat map showing the positive and negative correlations with GNG7 expression was obtained. Subsequently, GO functional annotation and KEGG enrichment analysis of GNG7-related genes were further performed.

## Immunomodulator

The TISIDB database (www.http://cis.hku.hk/TISIDB/) has calculated the associations between multiple genes and immune characteristics of 30 TCGA cancer types, such as lymphocytes, immunomodulators, and chemokines [35]. The data in this database come from 4176 unpublished records in 2530 individuals, and the publications report 988 genes related to anti-tumor immunity. High-throughput sequencing and genomic analysis data were used to identify genes related to tumor cell immune infiltration. Using P ≤ 0.05 as the threshold, immunoinhibitors and immunostimulators significantly related to GNG7 gene expression were obtained. Thereafter, these obtained immune modulators were uploaded to two web-based tools (https://string-db.org/, http://www.webgestalt.org/). Later, the interaction relationship between proteins, such as physical contact and targeted regulation, was visualized by constructing a protein–protein interaction (PPI) network diagram using the former tool [36]. While the latter tool was employed for KEGG pathway enrichment analysis on GNG7-related immune modulators [37].

## Survival analysis

This study aimed to develop a multiple immune gene signal based on the GNG7-related immunomodulators to predict prognosis. Cox regression, which is also referred to as Cox proportional hazard model, is an important method for survival analysis. British statisticians have linked death risk with related factors to construct a new formula, which is expressed as h(t) = h0(t)exp(β1 × 1 + β2 × 2 = …… + βjxj). Where h(t) stands for the risk function of the research object, which changes with time; × 1, × 2…xj are the independent variables, while β1, β2…, βj are the regression coefficients. Next, Kaplan–Meier (K-M) curves, together with univariate and multivariate Cox regression, were used to analyze the relationship between the clinical characteristics of patients and the overall survival (OS) rate of the disease in GNG7-related genes and clinical features. Finally, by combining the clinical characteristics and risk scores of patients, a nomogram was constructed to predict cancer prognosis [38].

## External validation

GSE40435(normal:101, tumor:101) was downloaded from GEO database. Each sample contains the amount of GNG7 expression and is used for differential analysis of GNG7 gene expression. Tissue sections were constructed by the Pathology Department of the Second Affiliated Hospital of Chongqing Medical University Paraffin sections were washed with xylene and deparaffinized and hydrated with graded ethanolic solutions. Afterwards, the sections were boiled in sodium citrate buffer, followed by dropwise addition of 3% hydrogen peroxide to inactivate tissue endogenous peroxidase. Then, the incubated and washed sections were incubated with polyclonal GNG7 antibody (A10690-1) overnight at 4 °C, and washed with a biotin-conjugated secondary antibody at 37 °C for 40 min. Sections were then stained with hematoxylin–eosin, and finally, photographs were taken under a light microscope [39].

## Statistical analysis

R software (version 3.6.3 and 4.1.1) was adopted for all statistical analyses. Differences between CCRCC and healthy tissues were determined using paired t-test and Mann–Whitney U test. The ggplot2 and survivalROC packages in R software were employed to visualize the differences between tissues and to construct the survival curves.

## Results

Here in this study, we first examined the oncogenic role of GNG7 in CCRCC, then further analyzed the relationship between the GNG7 gene and immune infiltrating cells in CCRCC and also discussed the relationship between GNG7-related genes in the immune function pathway in kidney cancer cell lines. To conclude, GNG7-related immunomodulators were identified through the TISIDB online website, and a prognostic prediction model for CCRCC patients was constructed based on key immune prognostic genes, and then the expression of GNG7 in CCRCC was validated using the GEO external validation set and immunohistochemical experiments.

### Differential expression and clinicopathological analysis of GNG7 in CCRCC and healthy tissues

**Fig. 2:**
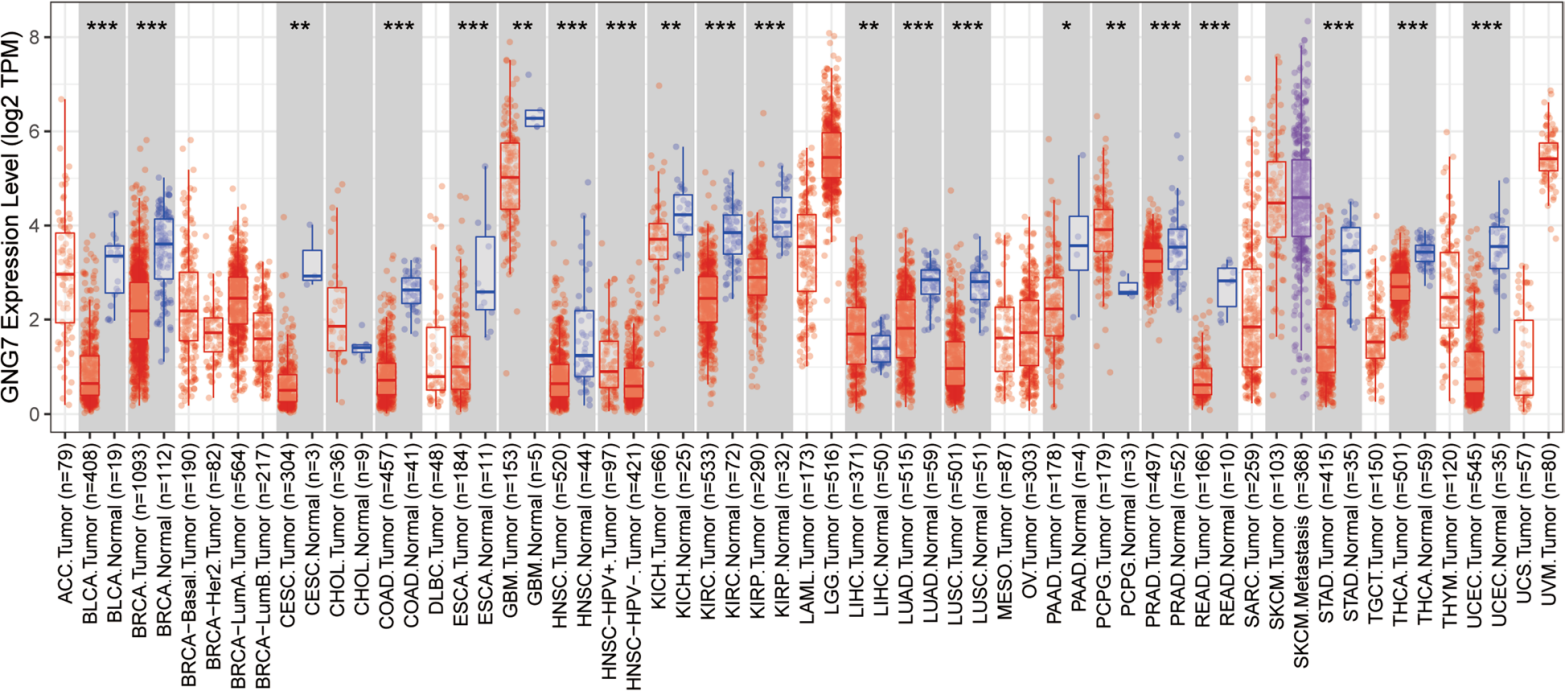
Expression pattern of GNG7 in Pan-cancer perspective. The mRNA expression of GNG7 was downregulated in 17 of 18 cancer types compared with normal tissues. Ns, no significance; *p < 0.05; **p < 0.01; ***p < 0.001

**Fig. 3:**
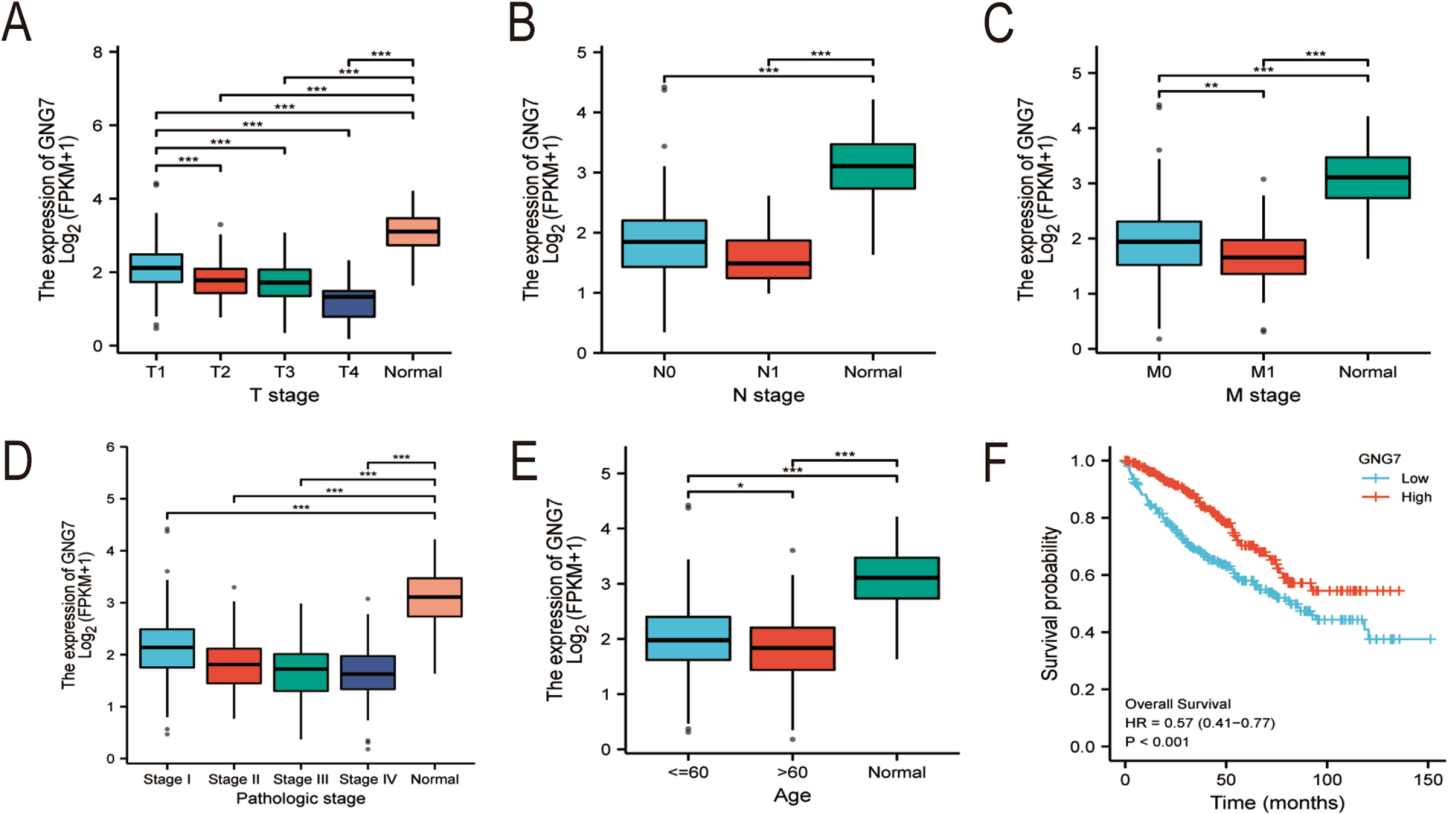
Relationships between GNG7 mRNA levels and clinical pathological characteristics and Kaplan–Meier curves forGNG7. GNG7 mRNA expression was significantly correlated with T stage (**A**), N stage (**B**), M stage (**C**), pathologic stage (**D**) and age (**E**). (ns, no significance, *P < 0.05, **P < 0.01, ***P < 0.001), (**F**) Kaplan–Meier survival curves indicated that CCRCC patients with low GNG7 mRNA expression had a shorter OS than those with high-level of GNG7 (P < 0.001)

The expression of GNG7 in pan-cancer was obtained from TIMER. It was observed from the results that, GNG7 was lowly expressed in most tumors and was significantly different from the expression in healthy tissues (Fig. 2). The expression of GNG7 in CCRCC was further explored based on TCGA database.Consistent with the findings of previous studies, GNG7 expression was significantly correlated between tumor samples and normal samples, including MRNA expression in unpaired and paired samples and protein expression in unpaired samples (Supplementary Figure S1A-C). Moreover, it was figured out based on clinicopathological analysis that, GNG7 expression gradually decreased with the increase in patient age and tumor stage (Fig. 3A, B, D, E). From the perspective of tumor staging, patients with distant metastasis also had lower GNG7 expression than those without distant metastasis (Fig. 3C). Combined with the above results, it was concluded that GNG7 mRNA and protein expression showed consistent attenuation in CCRCC tissues. Moreover, KM curve revealed that GNG7expression was significantly positively correlated with OS (Fig. 3F), and the down-regulated GNG7 expression was more obvious in advanced tumors.

### Immunohistochemistry and GEO database validation of GNG7 in CCRCC

**Fig. 4:**
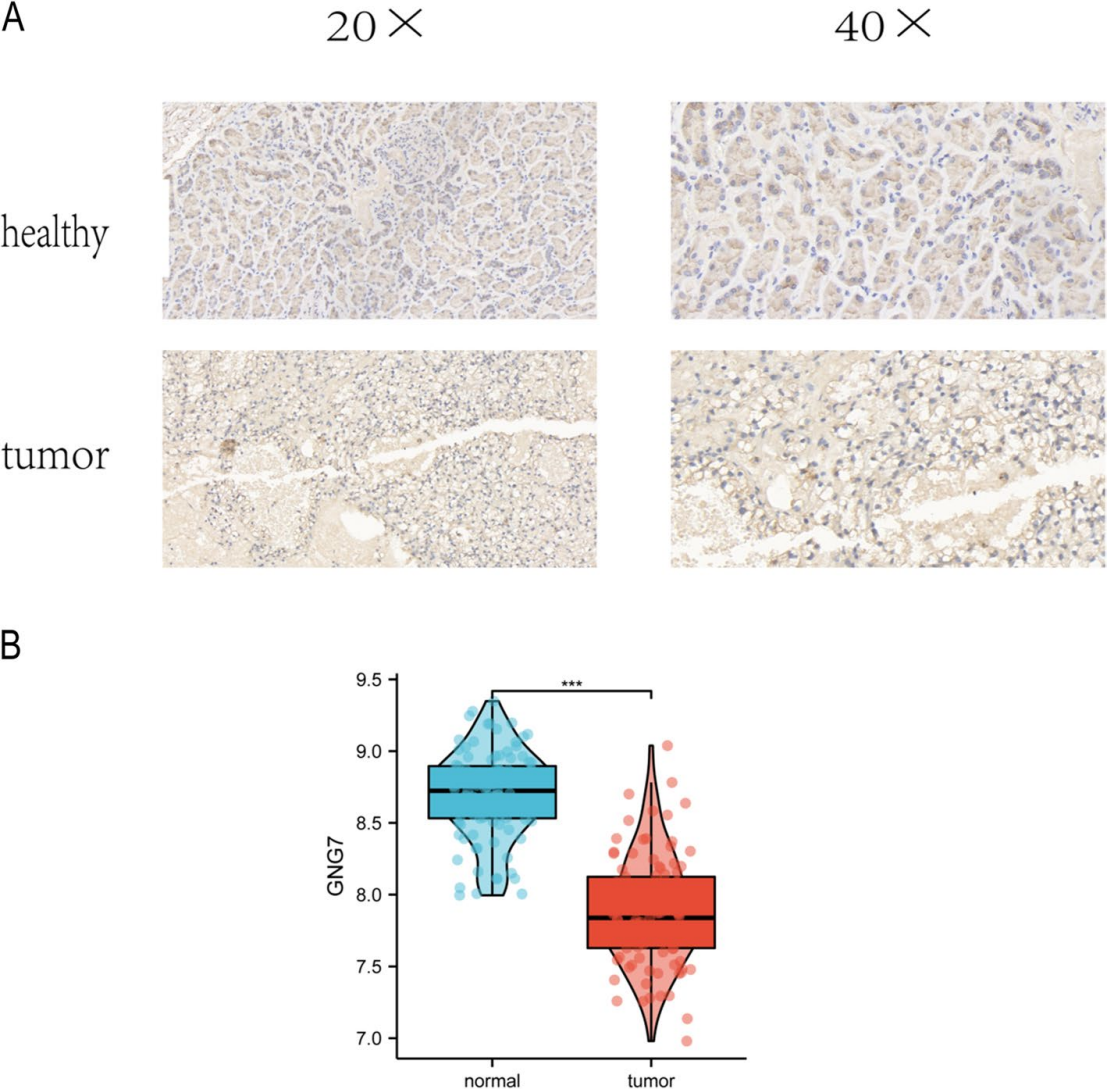
Validation of external GNG7 expression. **A** Immunohistochemical staining to detect the expression of GNG in kidney tissue. **B** Expression of GNG7 in kidney tissue in GSE40435

Immunohistochemistry (IHC) scores the specimens according to the staining intensity, and the staining intensity can be divided into 3 grades, grade 0 with no positive staining, grade 1 weakly positive in pale yellow, grade 2 moderately positive in tan, and grade 3 strongly positive in tan. We made a total of 5 specimens, including 3 renal clear cell carcinoma tissues and 2 adjacent tissues, and made two immunohistochemical sections for each specimen. Immunohistochemical results showed that GNG7 was positive in light yellow in adjacent tissues, while tumor tissues showed no positive staining (Fig. 4A). The expression of GNG7 in adjacent tissues was higher than that in the control group. The results show that GNG7 is low expressed in tumors and significantly different from that in normal tissues (Fig. 4B), which is consistent with our results.

### Immune cell infiltration in CCRCC and normal tissues

**Fig. 5:**
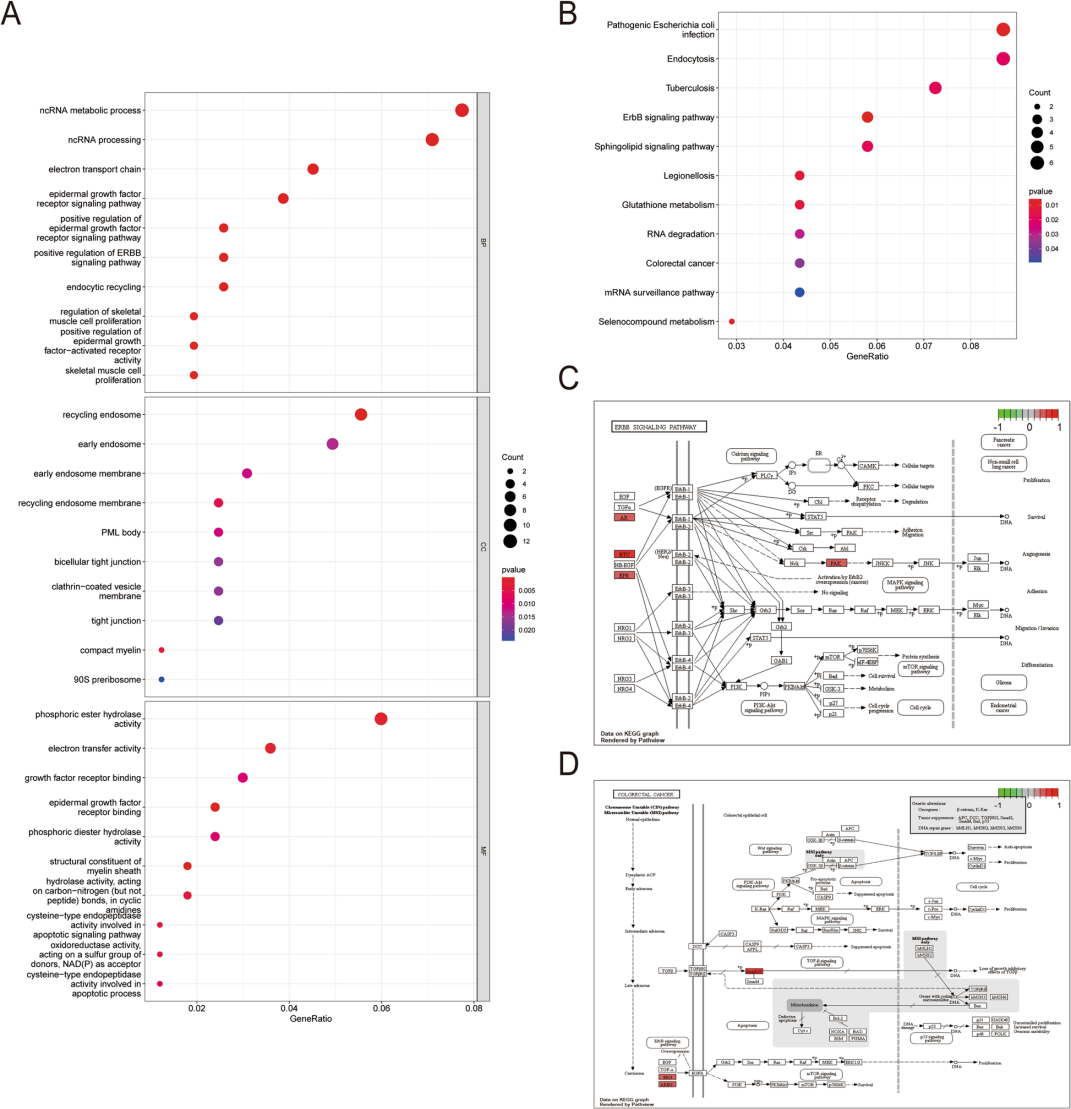
Gene functional enrichment analysis. (**A**) GO-term function enrichment analysis of GNG7-related genes. (**B**) KEGG-term function enrichment analysis of GNG7-related genes. Differentially expressed genes were involved in the ERBB signaling pathway (**C**) and the Colorectal cancer pathway (**D**)

At first, the characteristic gene expression profiles were extracted and processed by the CiberSort method to systematically describe the infiltration patterns of immune cells. Compared with healthy tissues, the ratios of T cells CD8, T cell CD4 naive, T cells follicular helper, T cells regulatory, Macrophages M0, and Macrophages M1 in CCRCC patients increased significantly, while those of B cells naive, plasma cells, and T cells CD4 memory resting, dendritic cells resting, and mast cells resting significantly reduced (Supplementary Figure S2A). Based on the 32 renal cancer cell lines in the CCLE database, 207 genes significantly related to GNG7 were obtained upon the threshold of *P* < 0.05. Accordingly, gene data positively and negatively related to GNG7 were obtained (Supplementary Figure S2B 2C). In addition, GO functional annotation was used to evaluate the molecular functions (MFs), biological processes (BPs) and cellular components (CCs) of these genes (Fig. 5A). Moreover, KEGG enrichment analysis of GNG7-related genes was conducted, which suggested that the ERBB signaling pathway was related to the immune status mediated by GNG7, and the Colorectal cancer pathway was associated with the tumor status mediated by GNG7 (Fig. 5B, C, D). The above results indicated that GNG7 might play a role in the tumor environment and immune regulation of CCRCC.

### The associations between GNG7 and immune cells

**Fig. 6:**
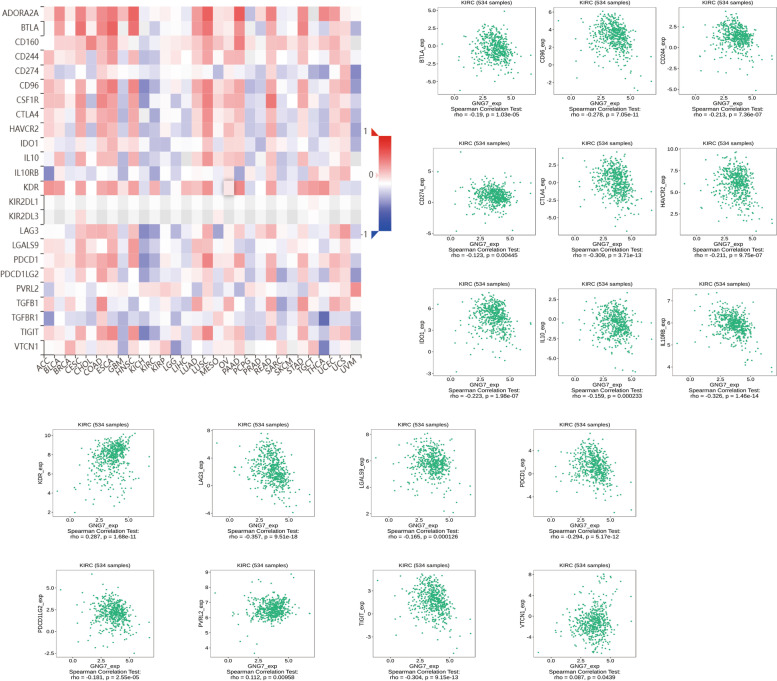
Correlation between GNG7 expression levels and Immunoinhibitors (Only expressions that differ from GNG7 will be displayed)

**Fig. 7:**
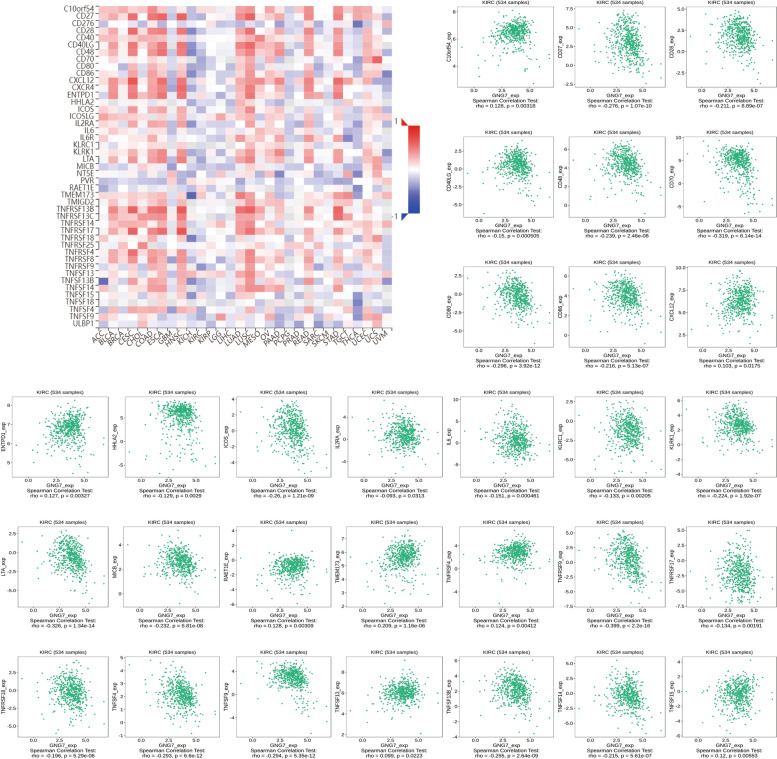
Correlation between GNG7 expression levels and Immunostimulators (Only expressions that differ from GNG7 will be displayed)

**Fig. 8:**
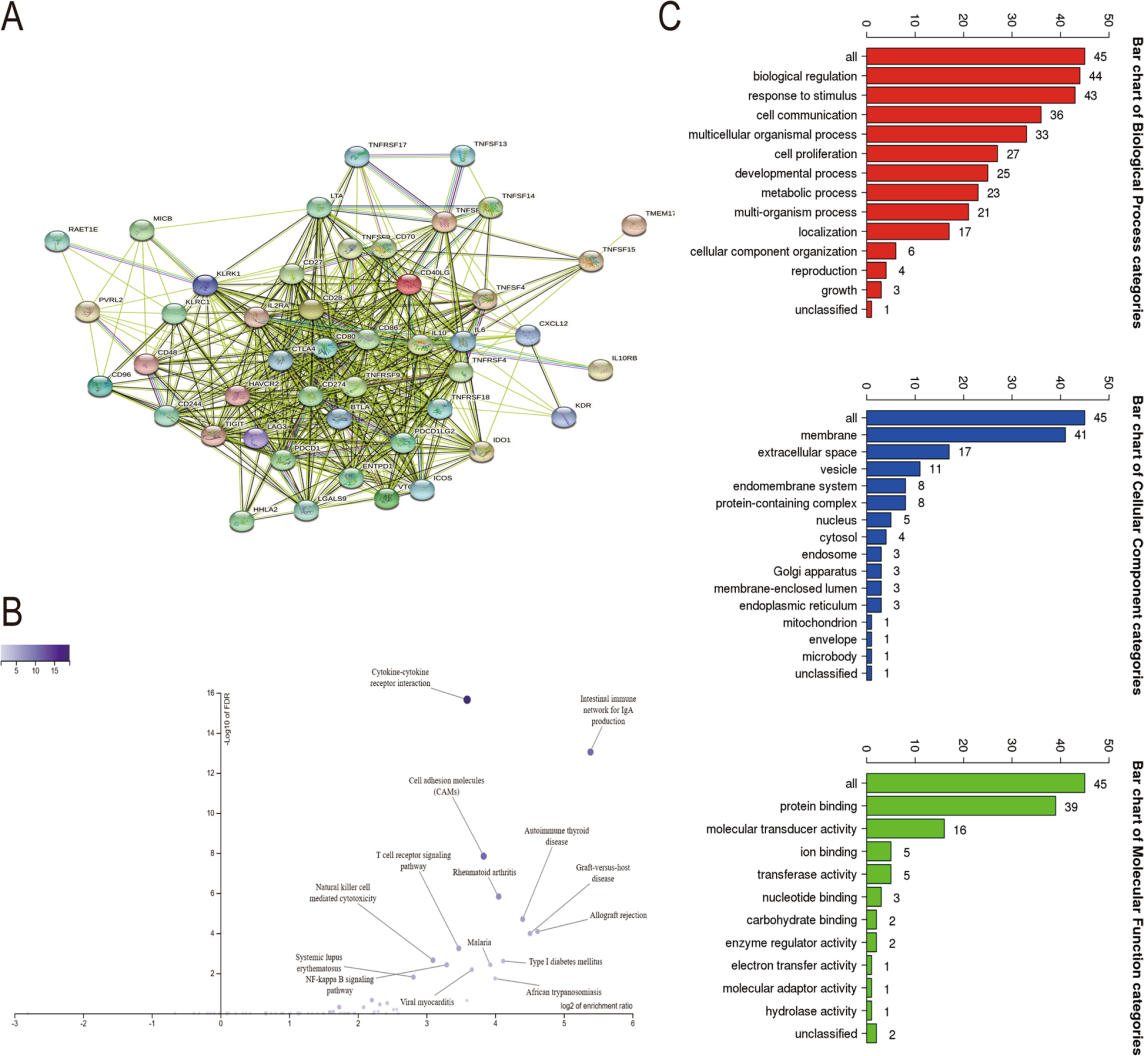
Identification and analysis of immunomodulators associated with the GNG7 gene. **A** Protein–protein network of 47 GNG7-associated immunomodulators in CCRCC, produced by the STRING online server. **B** Gene Ontology annotation of 47 GNG7-associated immunomodulators in CCRCC. **C** Kyoto Encyclopedia of Genes and Genomes pathway analysis of the abovementioned 47 genes

Next, GNG7 expression profiles in six tumor-infiltrating immune cells (TIICs) and four immunosuppressive cells were obtained based on the TIMER database to further investigate the effect of GNG7 on the immune system (Supplementary Figure S3A-B). The above results indicated that GNG7 might play a role in the immune mechanism of CCRCC. After defining the statistical significance as *p* ≤ 0.05, 17 Immunoinhibitors and 30 Immunostimulators were obtained from the TISIDB online website (Figs. 6 and 7). Thereafter, the PPI network diagram of GNG7-related immune modulators and the KEGG volcano diagram were plotted using the online tools (Fig. 8A, C) [40]. The credibility threshold of PPI mapping was set to 0.4. As shown by the KEGG volcano map, T cell receptor signaling pathway, Intestinal immune network for IgA production, Natural killer cell mediated cytotoxicity, Autoimmune thyroid disease and other pathways were related to the immune status mediated by GNG7-related immune modulators (Fig. 8B), which again verified that GNG7 might participate in and mediate the immune events of CCRCC.

### Prognostic significance of immunomodulators related to GNG7 in renal cell carcinoma

**Fig. 9:**
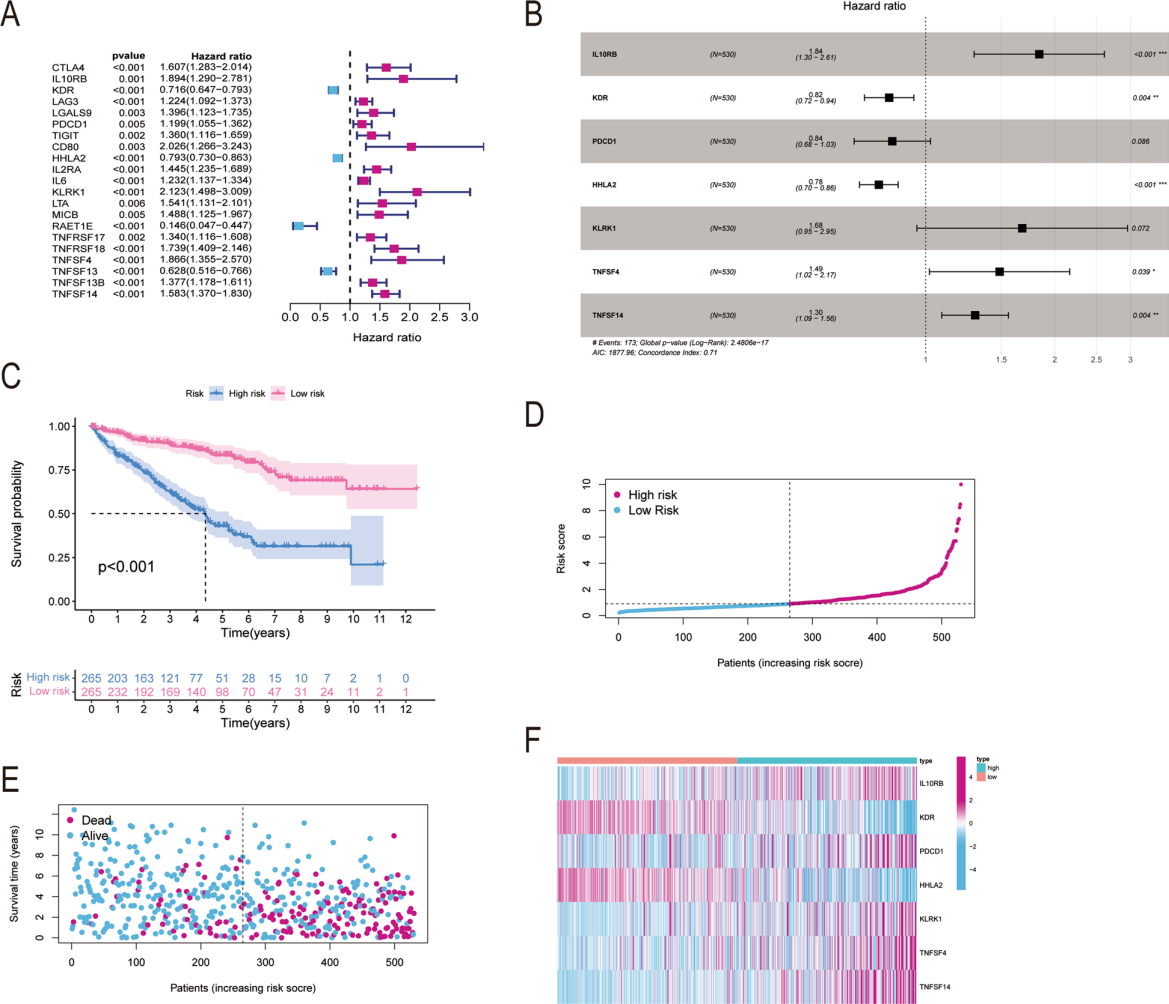
The development of prognostic gene signatures based on 47 GNG7-associated immunomodulators. The hazard ratios of genes integrated into the prognostic signatures are shown in the forest plots for CCRCC based on Univariate Cox regression analyses (**A**) and multivariate Cox regression analyses (**B**). Kaplan–Meier curves for CCRCC (**C**). Distribution of risk scores (**D**), along with survival statuses (**E**), and gene expression profiles (**F**) for CCRCC

**Fig. 10:**
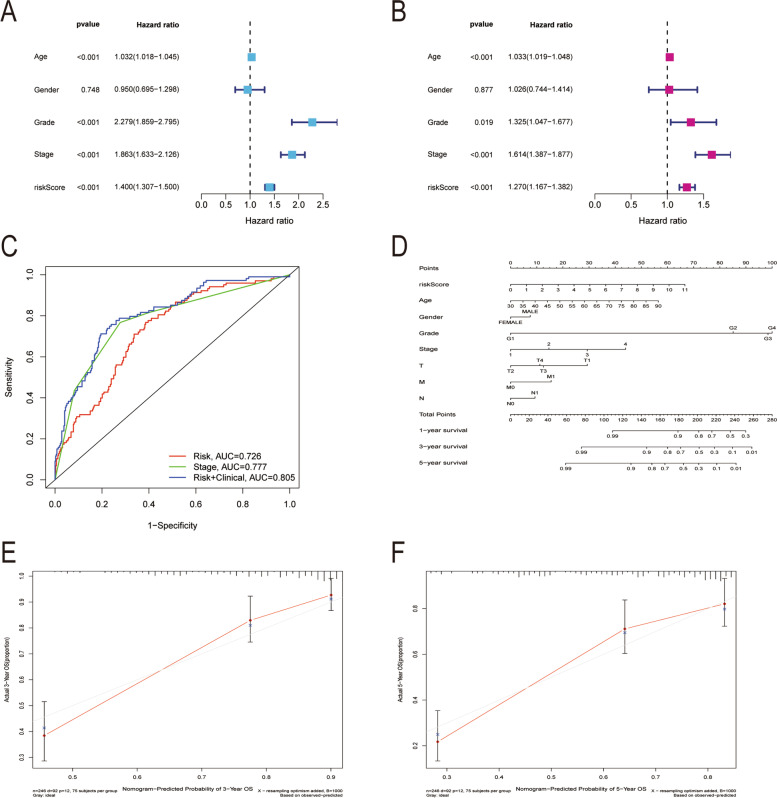
Construction of a GNG7-related immunomodulator risk model. Univariate (**A**) and multivariate (**B**) independent prognostic analysis of independent risk factors for OS in patients with ccRCC. (**C**) Time-independent ROC analysis of risk scores for OS prediction in the TCGA database. (**D**) A nomogram for predicting 1-,3- and 5-year survival possibilities of individual CCRCC patients. The calibration curve of 3-year (**E**) and 5-year survival of CCRCC patients (**F**). The 45◦ grey line represented a perfect uniformity between nomogram-predicted and real possibilities

The studied variables were subject to univariate and multivariate COX analyses, so as to explore the prognostic value of GNG7-related immunomodulators in CCRCC. Upon univariate Cox regression analysis on GNG7-related immune regulators, the top 21 prognostic genes in CCRCC were obtained, including 17 high-risk genes and 4 low-risk genes (*P* < 0.01) (Fig. 9A). Thereafter, the corresponding risk score of each sample was obtained by the sum of the product of gene expression and the correlation coefficient, and then the median risk score of all samples was calculated as the threshold to classify samples into high-risk and low-risk groups. Using this method, 7 immune genes related to the prognosis of CCRCC were obtained, including 4 high-risk and 3 low-risk genes (Fig. 9B, Table 1). Furthermore, the K-M survival curve demonstrated that patients with low-risk scores had significantly longer survival time than those with high-risk scores (log-rank test, statistical threshold *P* < 0.001, Fig. 9C). Also, the risk score chart showed that the risk score increased with the increase in risk, whereas the survival status chart indicated that patients with death risk were concentrated in the high-risk score area (Fig. 9D, E). The risk heat map clearly revealed that the risk scores of patients were significantly positively correlated with IL10RB, KLRK1, TNFSF4, and TNFSF14, but significantly negatively correlated with KDR, PDCD1, and HHLA2 (Fig. 9F). In the univariate Cox regression model, age (HR = 1.032, *P* < 0.001), stage (HR = 2.279, *P* < 0.001), clinical grade (HR = 1.863, *P* < 0.001) and risk score (HR = 1.400, *P* < 0.001) were significantly related to the survival rate of patients (Fig. 10A). In addition, by multivariate Cox regression analysis after adjusting for age, gender, grade, TMN stage, it was concluded that age (HR = 1.033, *P* < 0.001), stage (HR = 1.325, *P* = 0.019), clinical grade (HR = 1.614, *P* < 0.001) and risk score (HR = 1.382, *P* < 0.001) were the independent indicators for predicting the prognosis of CCRCC (Fig. 10B). Additionally, the ROC curve was plotted to verify the sensitivity of the model (Fig. 10C). Finally, the prognostic nomogram of CCRCC was obtained and validated by evaluating all the clinical information to predict the survival probability of an individual (Fig. 10D). As revealed by the calibration curve, the red line, which represented the model-predicted survival probability of an individual, was well matched with the grey line, which represented the ideal reference line for the 3-year and 5-year survival rates of an individual in the nomogram (Fig. 10E, F).

**Table 1:** Multifactorial analysis of correlation coefficients for seven genes

id	coef	HR	HR.95L	HR.95H	*p* value
IL10RB	0.609311627	1.839164931	1.297255271	2.607449527	0.000623313
KDR	-0.19567427	0.822280032	0.720010601	0.939075688	0.003882144
PDCD1	-0.179116853	0.836008204	0.681267736	1.025895811	0.086317322
HHLA2	-0.251343473	0.777755188	0.70110672	0.862783247	2.05E-06
KLRK1	0.518104765	1.678842832	0.954073361	2.954189236	0.072350516
TNFSF4	0.396494925	1.486604894	1.020342169	2.165934308	0.038938392
TNFSF14	0.265337306	1.303870705	1.090234608	1.559369702	0.003658256

## Discussion

Tumor immunity includes antigen presentation, T cell activation, avoidance of local suppression and tumor cell killing, which is a complex process [41]. Failure of each of these steps may reduce the efficacy of immunotherapy and cause incalculable harm. Therefore, the appropriate choice of immunotherapy is a major challenge in oncology treatment. Biomarkers that reflect the efficacy of immunotherapy and the prognosis of tumor patients are very important for treatment decisions in CCRCC.

GNG7 (G protein γ subunit 7) is a subunit of heterotrimeric G protein [42]. Previous experiments have shown that GNG7 expression reduces tumor volume in mice [23]. GNG7 is the first autophagy-inducing regulator of Gγ protein reported [43]. A study shows that low expression of GNG7 may induce the proliferation of LUAD tumor cells, increase the probability of invasion in vitro, and promote tumor growth and development in vivo [44]. In addition, GNG7 inhibits cell division by regulating the actin cytoskeleton, and it shows low expression in many tumors [19–23, 43, 45]. Ohta et al*.* reported that the expression of GNG7 in esophageal cancer was extremely suppressed compared with that in normal tissues. Such inhibition is related to tumor invasion and disease prognosis and is regulated by miRNAs. However, the GNG7 expression-induced progression of esophageal cancer may be attributed to the hypermethylation of GNG7 promoter [24]. In addition, Hartmann et al*.* also drew the same conclusion in head and neck tumors that the expression of GNG7 was related to tumor size. They also found that the expression of GNG7 protein was significantly related to tumor keratinization, and the lack of keratinization usually indicates tumor progression [6]. Recent studies suggested that GNG7 is highly associated with the B-cell receptor signaling pathway. Low levels of B-cell infiltration in patients with low GNG7 expression often suggest a poorer prognosis [46].

Consistent with the results of previous studies, our results showed that GNG7 mRNA and protein expression was down-regulated in CCRCC tissues, and the down-regulation degree was extremely related to tumor grade. K-M curve survival analysis verified that GNG7 down-regulation was significantly related to high mortality. Multivariate Cox analysis indicated that the expression of GNG7 served as an independent risk factor for the OS of CCRCC. Moreover, the tumor prognostic model constructed based on GNG7-related immunomodulators was able to predict the 3-year and 5-year survival of CCRCC. In addition, the model analysis curve was well matched with the standard curve of CCRCC survival. Based on previous studies and this research, it was reasonably speculated that GNG7 predicted the diagnosis and prognosis of CCRCC well, and it was also used as an important evaluation factor for tumor recurrence and early metastasis.

In the presented study, we used COX regression analysis to obtain 7 immune regulators associated with GNG7, after deriving 7 immune genes based on further risk scoring. Among them, KDR, PDCD1, and HHLA2 are prognostic protective genes for CCRCC, in contrast to IL10RB, KLRK1, TNFSF4, and TNFSF14, which are risk factors for prognosis in CCRCC. SU et al. concluded by sequencing that epigenetic silencing of the KDR gene could affect the methylation of CCRCC and thus the tumor invasion [47]. Xiang et al. concluded by weighted gene co-expression network analysis that PDCD1 is a prognosis-related gene in CCRCC and is significantly associated with T-cell CD8, monocyte and mast cell resting [48]. Zhou et al. determined the gene expression level by immunohistochemistry of CCRCC cancer tissues and obtained that HHLA2 gene expression was higher than PD-1 in CCRCC, and HHLA2/PD-1 co-expression was significantly associated with high density CD8 + and CD4 + [49]. Jiang et al. [50] and He et al. [51] identified IL10RB and TNFSF14 as prognosis-related genes in CCRCC and could affect the tumor microenvironment of CCRCC by calculating immune scores, respectively. However, differing from previous studies, we combined the genes associated with GNG7 in kidney cancer cell lines and further elaborated the potential mechanisms and immune characteristics of these genes in kidney cancer by GO and KEGG gene enrichment analysis. We further elaborated the close relationship between 7 immunoprognostic genes and GNG7, and calculated the risk scores of these genes and combined them with clinical information to construct a model that can assess the prognosis of kidney cancer.

Tumor onset and development often begin with changes in the characteristics of tumor-surrounding matrix [52]. As an important part of tissue matrix, immune cells participate in driving and escaping disease development [9]. Before this, immune cells have been confirmed to exhibit a unique distribution and infiltration ratio in the tumor microenvironment (TME), and immunotherapy has gradually become an alternative treatment for many cancer patients [53]. The immune cell characteristics in CCRCC are manifested by infiltration of a variety of T cells, including CD8 + T cells and CD4 + T cells [54, 55]. The prognosis of CCRCC patients can be predicted clinically based on the infiltration patterns of different immune cells. Liu et al*.* found that GNG7 served as the autophagy-inducing gene mainly because it interacts with and inhibits the mTOR1 signaling pathway, and comprehensively suppresses the growth of effector cells by inducing autophagic cell death and cell division inhibition [43]. Therefore, it is reasonably speculated that GNG7 may be involved in the progression, metastasis, immune control, and drug resistance of CCRCC. In this study, we found that GNG7 was tightly related to TIICs. Previous reports indicate that high CD8 + T cell infiltration is associated with poor prognosis of CCRCC [54, 55]. Our research also discovered that GNG7 was negatively correlated with CD8 + T cell infiltration. Such result seems to further prove that GNG7, a tumor suppressor gene, is involved in tumor progression to some extent in the immune mechanism of tumors.

Furthermore, GNG7-related immunomodulators were used as the research object and a prognostic model for CCRCC was established through multivariate analysis. According to our results, the model was well matched with the actual survival time. According to the above results, we can construct a prognostic model for CCRCC based on the immune infiltration characteristics, and the expression of GNG7 and the characteristics of related TIICs can serve as the prognostic biomarkers and immunotherapeutic targets for CCRCC.

## Conclusions

In summary, our study suggests that low expression of GNG7 is strongly associated with immune cell infiltration patterns in CCRCC tumors. GNG7 may function as a tumor suppressor gene through immune pathways in CCRCC. We have developed a prognostic prediction model based on GNG7-related immunomodulators to forecast CCRCC patient outcomes. GNG7 shows promise as a potential therapeutic target for CCRCC. However, further validation through clinical trials and experimental studies is warranted to fully assess its clinical significance.

### Supplementary Information

**Additional file 1:** **Figure S1. **The mRNA and protein expression of GNG7 in CCRCC.(A) The mRNA expression levels of GNG7 in 72 CCRCC and matched-adjacent normal samples. (B) The mRNA expression levels of CCRCC in 539 CCRCC samples and 72 normal samples. (C) The protein expression levels of GNG7 based on CPTAC. (ns, no significance, *P < 0.05, **P < 0.01, ***P < 0.001).

**Additional file 2:** **Figure S2.** Correlation of immune cell infiltration and the heatmaps of differentially expressed genes (DEGs) regulated by GNG7 in CCRCC. (A) Violin plot visualizing the difference in immune infiltration between normal and tumor tissues. Gene data related to GNG7 positively (B) and negatively (C) can be obtained. (*P < 0.05, **P < 0.01, ***P < 0.001).

**Additional file 3:** **Figure 3.** The relationship between GNG7 expression and tumor immune cell infiltration. (A) Relationships between the GNG7 expression and six tumor-infiltrating immune cells. (B) Relationships between the GNG7 expression and 4 types of immunosuppressive cells.

## Data Availability

Publicly available datasets were analyzed in this study. This data can be found here: TCGA database (https://portal.gdc.cancer.gov/) and GEO database (https://www.ncbi.nlm.nih.gov/geo/).
