# Supplementary material for: Establishment of a new prognostic risk model of GNG7 pathway-related molecules in clear cell renal cell carcinoma based on immunomodulators
Source: BMC Cancer. 2023 Sep 13;23:864. doi: 10.1186/s12885-023-11265-8 (PMC10500784; doi:10.1186/s12885-023-11265-8)
Supplement: Supplementary file 1 — Additional file 1: Figure S1. The mRNA and protein expression of GNG7 in CCRCC.(A) The mRNA expression levels of GNG7 in 72 CCRCC and matched-adjacent normal samples. (B) The mRNA expression levels of CCRCC in 539 CCRCC samples and 72 normal samples. (C) The protein expression levels of GNG7 based on CPTAC. (ns, no significance, *P < 0.05, **P < 0.01, ***P < 0.001). [file 12885_2023_11265_MOESM1_ESM.docx]

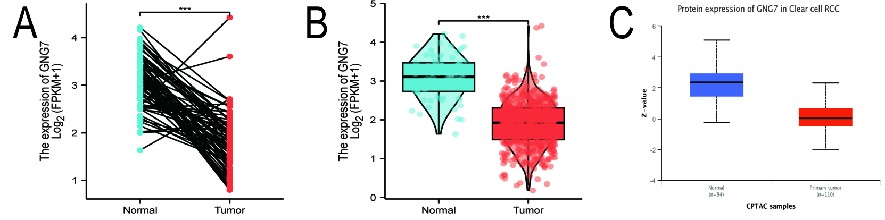


**(Supplementary Figure 1) The mRNA and protein expression of GNG7 in CCRCC.** (A) The mRNA expression levels of GNG7 in 72 CCRCC and matched-adjacent normal samples. (B) The mRNA expression levels of CCRCC in 539 CCRCC samples and 72 normal samples. (C) The protein expression levels of GNG7 based on CPTAC. (ns, no significance, *P < 0.05, **P < 0.01, ***P < 0.001).
