# Supplementary material for: Establishment of a new prognostic risk model of GNG7 pathway-related molecules in clear cell renal cell carcinoma based on immunomodulators
Source: BMC Cancer. 2023 Sep 13;23:864. doi: 10.1186/s12885-023-11265-8 (PMC10500784; doi:10.1186/s12885-023-11265-8)
Supplement: Supplementary file 2 — Additional file 2: Figure S2. Correlation of immune cell infiltration and the heatmaps of differentially expressed genes (DEGs) regulated by GNG7 in CCRCC. (A) Violin plot visualizing the difference in immune infiltration between normal and tumor tissues. Gene data related to GNG7 positively (B) and negatively (C) can be obtained. (*P < 0.05, **P < 0.01, ***P < 0.001). [file 12885_2023_11265_MOESM2_ESM.docx]

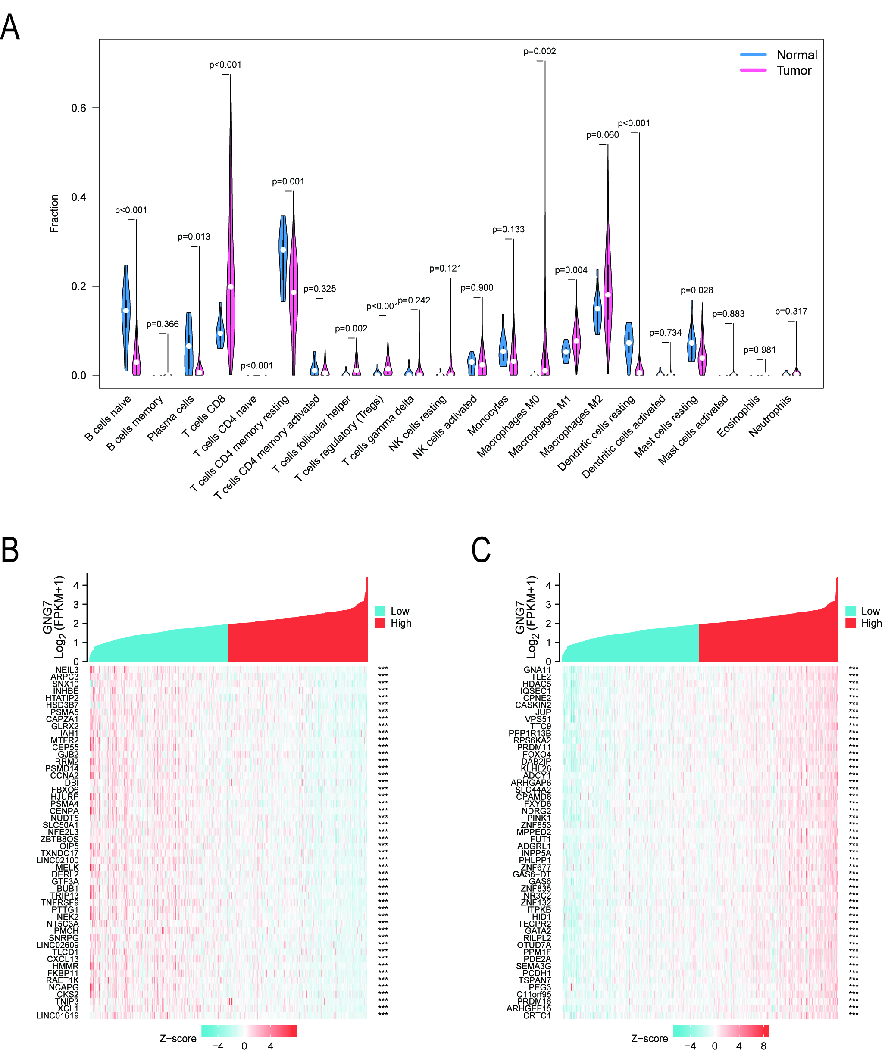


**(Supplementary Figure2) Correlation of immune cell infiltration and the heatmaps of differentially expressed genes (DEGs) regulated by GNG7 in CCRCC.** (A) Violin plot visualizing the difference in immune infiltration between normal and tumor tissues. Gene data related to GNG7 positively(B) and negatively(C) can be obtained. (*P < 0.05, **P < 0.01, ***P < 0.001)
