# Supplementary material for: Establishment of a new prognostic risk model of GNG7 pathway-related molecules in clear cell renal cell carcinoma based on immunomodulators
Source: BMC Cancer. 2023 Sep 13;23:864. doi: 10.1186/s12885-023-11265-8 (PMC10500784; doi:10.1186/s12885-023-11265-8)
Supplement: Supplementary file 3 — Additional file 3: Figure 3. The relationship between GNG7 expression and tumor immune cell infiltration. (A) Relationships between the GNG7 expression and six tumor-infiltrating immune cells. (B) Relationships between the GNG7 expression and 4 types of immunosuppressive cells. [file 12885_2023_11265_MOESM3_ESM.docx]

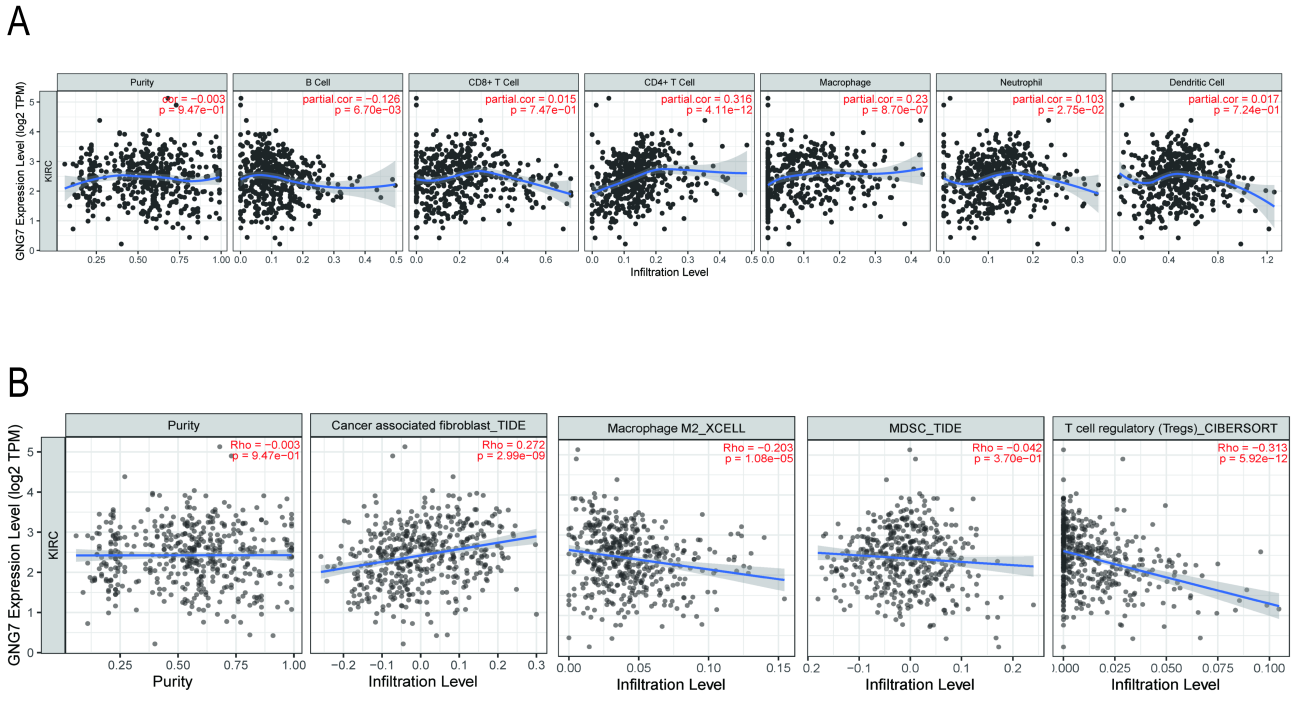


**(Supplementary Figure3) The relationship between GNG7 expression and tumor immune cell infiltration.** (A)Relationships between the GNG7 expression and six tumor-infiltrating immune cells. (B)Relationships between the GNG7 expression and 4 types of immunosuppressive cells.
